# Supplementary material for: Sexual behavior is linked to changes in gut microbiome and systemic inflammation that lead to HIV-1 infection in men who have sex with men
Source: Commun Biol. 2024 Sep 14;7:1145. doi: 10.1038/s42003-024-06816-z (PMC11401892; doi:10.1038/s42003-024-06816-z)
Supplement: Supplementary file 2 — Supplementary Information [file 42003_2024_6816_MOESM2_ESM.pdf]

Contents

1 Supplementary Methods **2**
1.1 Introduction of natural effect models 2

2 Supplementary Figures **4**

3 Supplementary Tables **10**

1 Supplementary Methods

1.1 Introduction of natural effect models

Below, we provide a concise overview of the Natural Effect Models [1, 2, 3, 4] that have been implemented in this study. Let $Y_i(x, m)$ denote the potential outcome for subject i that had been observed if, possibly contrary to the fact, i had been assigned to exposure level x and mediator value m.

Definition 1 (Natural Direct Effect). Based on the nested counterfactuals, $Y\left(x, M\left(x^*\right)\right)$, the natural direct effect

$$\mathrm{NDE}=E\left\{Y\left(x_2, M\left(x^*\right)\right)-Y\left(x_1, M\left(x^*\right)\right)\right\}$$

expresses the expected exposure-induced change in outcome when the exposure level changes from x_1 to x_2 while keeping the mediator fixed at the value that had naturally been observed if the exposure level equal to x^*.

Definition 2 (Natural Indirect Effect). The natural indirect effect

$$\mathrm{NIE}=E\left\{Y\left(x^*, M\left(x_2\right)\right)-Y\left(x^*, M\left(x_1\right)\right)\right\}$$

reflects the expected difference in outcome if all subjects were exposed to same level x^*, but their mediator value had changed from the value it would take if exposed to x_1 to the value it would take if exposed to x_2.

Definition 3 (Natural Effect Models). Natural effect models are conditional mean models for nested counterfactuals $Y\left(x, M\left(x^*\right)\right)$:

$$E\left\{Y\left(x, M\left(x^*\right)\right) \mid C\right\}=g^{-1}\left\{\beta^T W\left(x, x^*, C\right)\right\}$$

with $g(\cdot)$ a known link function (e.g., the identity or logit link), C is a given set of observed baseline covariates, $W\left(x, x^*, C\right)$ a known vector with components that may depend on x, x^* and C, and β a vector including parameters that encode the natural effects of interest. It can, for instance, easily be inferred that in model:

$$E\left\{Y\left(x, M\left(x^*\right)\right) \mid C\right\}=\beta_0+\beta_1 x+\beta_2 x^*+\beta_3 C$$

β_1 captures the natural direct effect whereas β_2 captures the natural indirect effect, both corresponding to a one-unit increase in the exposure level.

Example (The Natural Effect Models Used in the Paper). In this paper, we have a binary outcome (SC vs. NC) and categorical exposure (Group 1 to Group 4). The natural effects model employed are logistic models:

$$\begin{aligned}
&\operatorname{logit} \operatorname{Pr}\left(Y\left(x, M\left(x^*\right)\right) \mid C\right) \\
&=\beta_0+\beta_{11} I(x= \text{ Group } 2)+\beta_{12} I(x= \text{ Group } 3)+\beta_{13} I(x= \text{ Group } 4) \\
&\quad+\beta_{21} I\left(x^*= \text{ Group } 2\right)+\beta_{22} I\left(x^*= \text{ Group } 3\right)+\beta_{23} I\left(x^*= \text{ Group } 4\right)+\beta_3 C
\end{aligned}$$

with x and x^* corresponding to hypothetical levels of the exposure, $M\left(x^*\right)$ to the level of mediator that would have been if the exposure was set to x^*, and $Y\left(x, M\left(x^*\right)\right)$ to the outcome

that would have been observed if the exposure was set to x and the mediator was set to $M(x^*)$. Exponentiating the model parameter estimates provides estimates that can be interpreted as odds ratios.

$$\mathrm{OR}^{\mathrm{NDE}}_{\mathrm{Group2\ vs.1}\ |C} = \frac{\mathrm{odds}\left\{Y\left(x = \mathrm{Group\,2}, M\left(x^*\right)\right) \mid C\right\}}{\mathrm{odds}\left\{Y\left(x = \mathrm{Group\,1}, M\left(x^*\right)\right) \mid C\right\}} = \exp\left(\beta_{11}\right)$$

$$\mathrm{OR}^{\mathrm{NDE}}_{\mathrm{Group3vs.1}|C} = \frac{\mathrm{odds}\left\{Y\left(x = \mathrm{Group\,3}, M\left(x^*\right)\right) \mid C\right\}}{\mathrm{odds}\left\{Y\left(x = \mathrm{Group\,1}, M\left(x^*\right)\right) \mid C\right\}} = \exp\left(\beta_{12}\right)$$

$$\mathrm{OR}^{\mathrm{NDE}}_{\mathrm{Group4\ vs.1}\ |C} = \frac{\mathrm{odds}\left\{Y\left(x = \mathrm{Group\,4}, M\left(x^*\right)\right) \mid C\right\}}{\mathrm{odds}\left\{Y\left(x = \mathrm{Group\,1}, M\left(x^*\right)\right) \mid C\right\}} = \exp\left(\beta_{13}\right)$$

$$\mathrm{OR}^{\mathrm{NIE}}_{\mathrm{Group2\ vs.1}\ |C} = \frac{\mathrm{odds}\left\{Y\left(x, M\left(x^* = \mathrm{Group\,2}\right)\right) \mid C\right\}}{\mathrm{odds}\left\{Y\left(x, M\left(x^* = \mathrm{Group\,1}\right)\right) \mid C\right\}} = \exp\left(\beta_{21}\right)$$

$$\mathrm{OR}^{\mathrm{NIE}}_{\mathrm{Group3\ vs.1}\ |C} = \frac{\mathrm{odds}\left\{Y\left(x, M\left(x^* = \mathrm{Group\,3}\right)\right) \mid C\right\}}{\mathrm{odds}\left\{Y\left(x, M\left(x^* = \mathrm{Group\,1}\right)\right) \mid C\right\}} = \exp\left(\beta_{22}\right)$$

$$\mathrm{OR}^{\mathrm{NIE}}_{\mathrm{Group4\ vs.1}\ |C} = \frac{\mathrm{odds}\left\{Y\left(x, M\left(x^* = \mathrm{Group\,4}\right)\right) \mid C\right\}}{\mathrm{odds}\left\{Y\left(x, M\left(x^* = \mathrm{Group\,1}\right)\right) \mid C\right\}} = \exp\left(\beta_{23}\right)$$

Assumptions of Natural Effect Models. The identification of natural effects relies on rather strong causal assumptions [5, 6]. Upon adjustment for a given set of observed baseline covariates C, it is deemed sufficient to control for confounding (i) between exposure and outcome, thereby satisfying

$$Y(x, m) \perp X \mid C \quad \text{for all levels of } x \text{ and } m,$$

(ii) between exposure and mediator, thereby satisfying

$$M(x) \perp X \mid C \quad \text{for all levels of } x,$$

(iii) between the mediator and outcome (after adjustment for the exposure), thereby satisfying

$$Y(x, m) \perp M \mid X = x, C \quad \text{for all levels of } x \text{ and } m,$$

The identification of natural effects requires the further 'cross-worlds perpendence' assumption

$$Y(x, m) \perp M\left(x^*\right) \mid C \quad \text{for all levels of } x, x^* \text{ and } m.$$

Fitting Natural Effect Models. The natural effect models in this paper were fitted used imputation-based approach. The imputation-based approach is carried out in two steps: 1) a working model is used to handle missingness in the outcome by estimating the outcome mean, 2) the natural effect model is fitted to the imputed data. The working model for the outcome mean $E(Y \mid X, M, C)$ is:

$$\begin{aligned} E\left\{Y\left(x, M\left(x^*\right)\right) \mid C\right\} &= \sum_m E(Y \mid X = x, M = m, C) \Pr\left(M = m \mid X = x^*, C\right) \\ &= E\left\{E(Y \mid X = x, M, C) \mid X = x^*, C\right\}. \end{aligned}$$

After fitting the working model, missing values in the original data are imputed, resulting in an expanded dataset. We can then obtain estimates of direct and indirect effects by applying a natural effect model to this expanded dataset. Of note is that the two-step approach used in natural effect models is distinct from the product method, also known as the product-of-coefficients method [7]. The latter involves two separate regressions: one fits the outcome based on the exposure, the mediator, and covariates, while the other fits the mediator based on the exposure and covariates.

2 Supplementary Figures

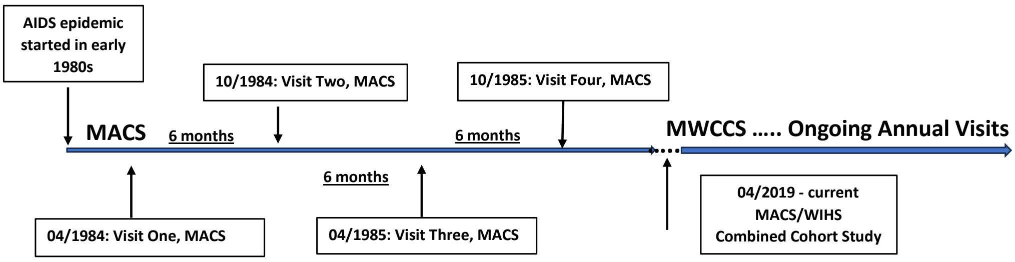

Supplementary Figure 1: **Information on this HIV-1 study cohort and study participants.** At visit one, demographic information (age, gender, race) was collected; At visits one to four, personal information, including sexual activity, alcohol usage, substance use, was collected. At visit one through visit three, blood, oral wash, urine, semen and stool samples were collected from study participants. MACS: Multicenter AIDS Cohort Study, WIHS: Women's Interagency HIV Study, MWCCS: MACS-WIHS Combined Cohort Study.

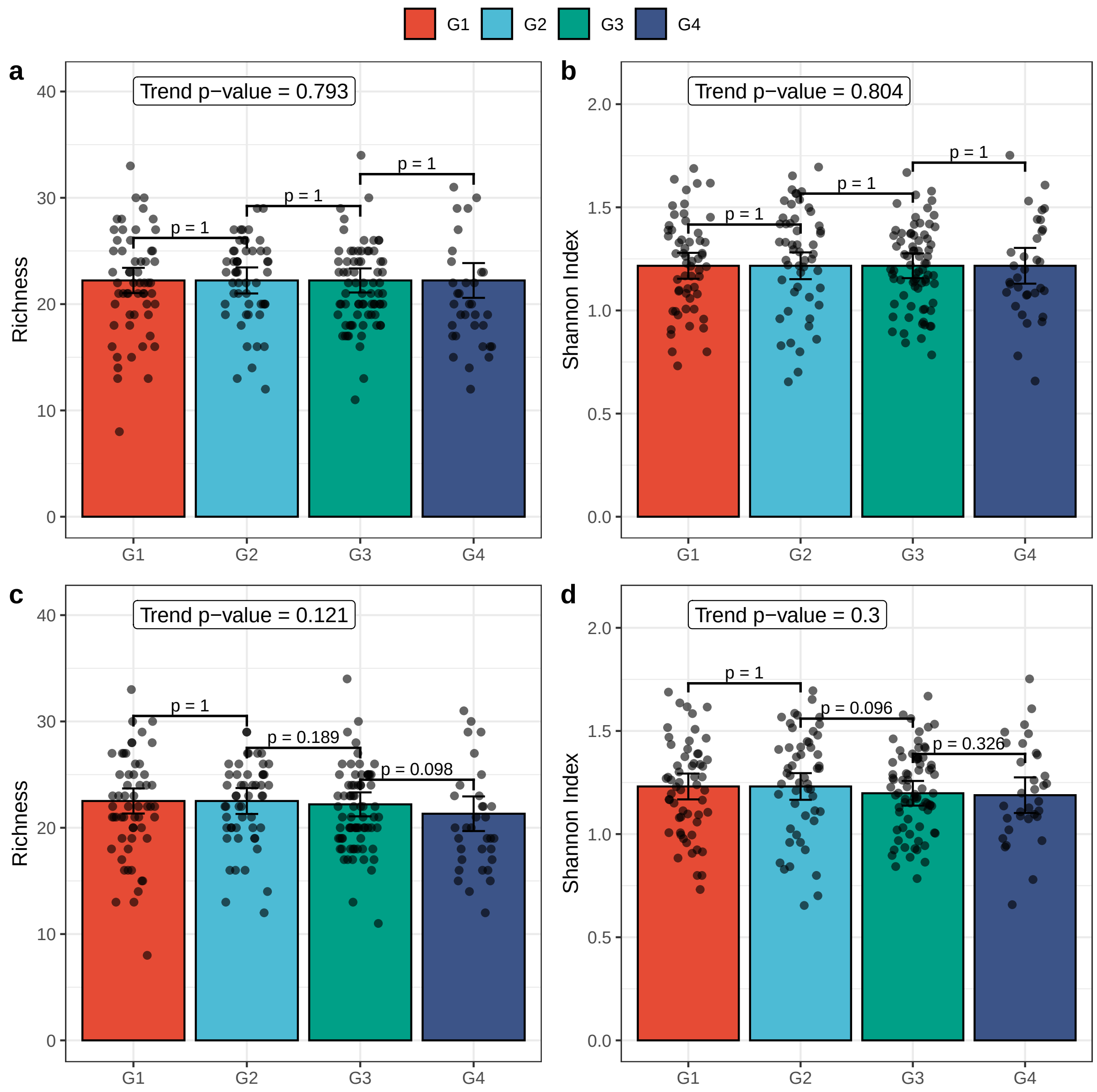

Supplementary Figure 2: **Bar plots showcase monotonically increasing or decreasing trend between exposure groups and microbial alpha diversities at the species level.** (a) species richness with an increasing trend, (b) Shannon diversity index with an increasing trend, (c) species richness with a decreasing trend, and (d) Shannon diversity index with a decreasing trend. The X-axis defines the exposure groups, spanning from Group 1 (G1) to Group 4 (G4). The Y-axis indicates the alpha diversity's effect size, either richness or Shannon index, as determined by the constrained linear mixed effects (CLME) model. It is important to note that these are not raw abundances but fitted values under monotonic trend. Error bars on each bar represent the 95% confidence interval (CI). Pairwise p-values (one-sided), contrasting the exposure groups, are illustrated above the brackets encompassing the corresponding bars. The plot includes the overall p-value for the monotonic trend evaluation. Notably, the analyses revealed no significant associations with the alpha diversity metrics.

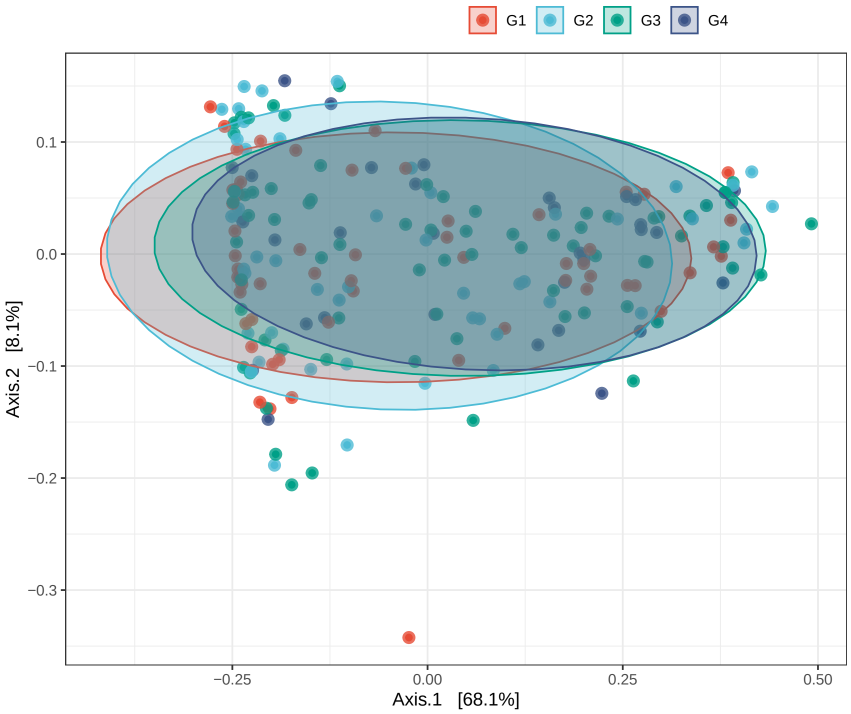

X	Y	p	q
G1	G2	0.66	0.66
G1	G3	0.07	0.13
G1	G4	0.09	0.13

Supplementary Figure 3: **PCoA plot of the microbial beta diversity (Bray-Curtis dissimilarity) at the species level.** Each point corresponds to an individual participant, color-coded by their exposure groups. Ellipses cover 80% of the data distribution. Adjacent tables detail contrasts, p-values (two-sided) ascertained through pairwise Permutational Multivariate Analysis of Variance (PERMANOVA), and adjusted p-values (q-values) that are rectified utilizing the Benjamini-Hochberg (BH) procedure.

a

b

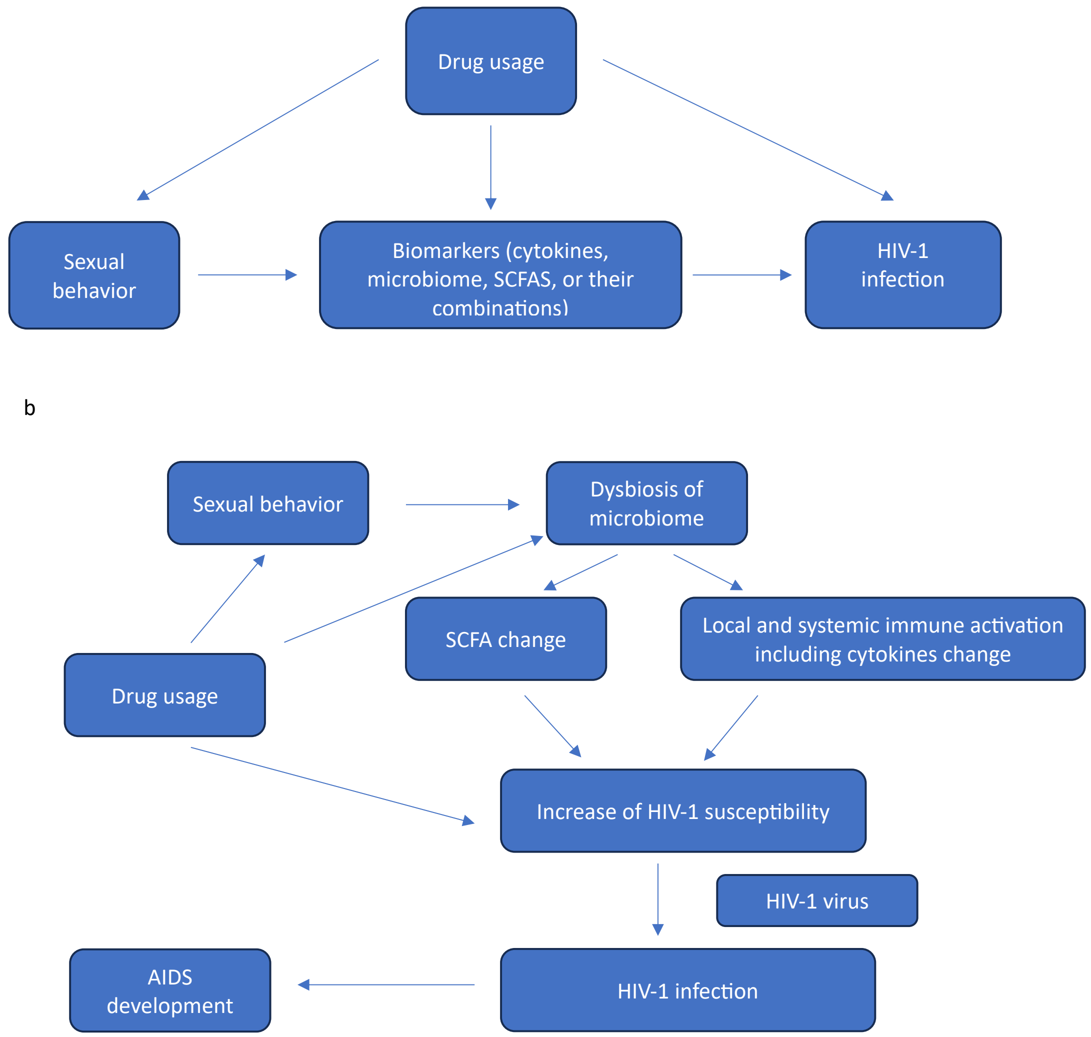

Supplementary Figure 4: **Directed Acyclic Graph (DAG) depicting the mediation analyses.** (a) Represents the working model employed in the current study, while (b) Illustrates a theoretically robust model that we proposed. However, the latter was not implemented in this paper due to constraints in the statistical methodology.

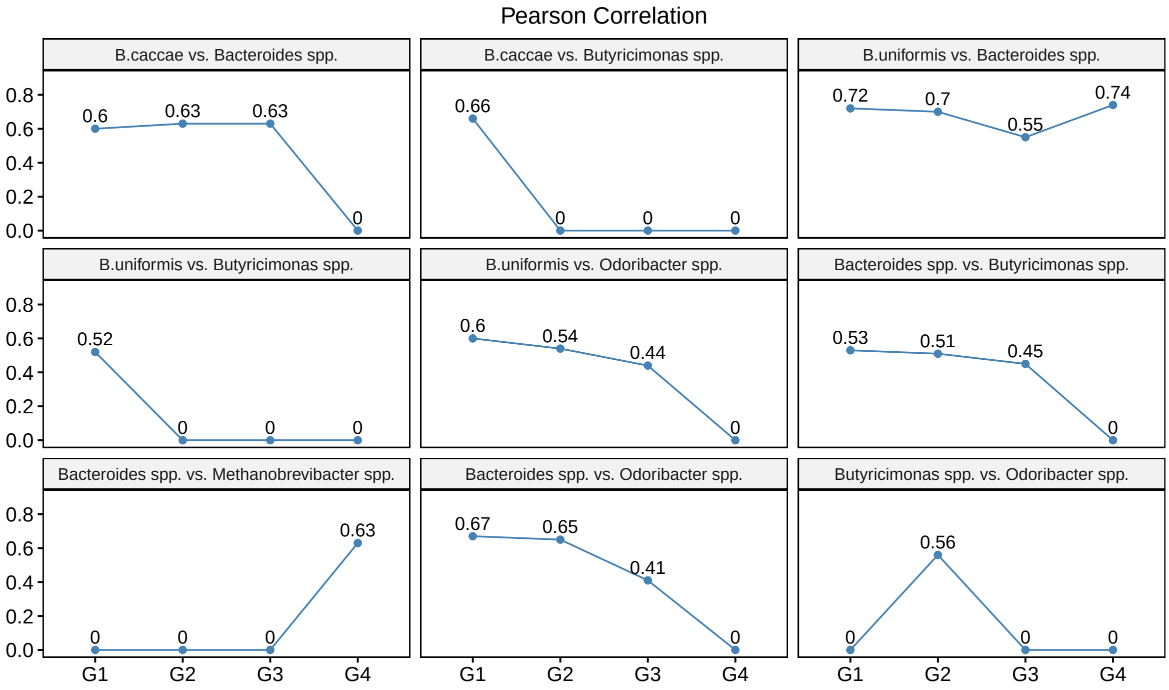

Supplementary Figure 5: **SECOM results of pairwise Pearson correlation coefficients between differentially abundant species.** The X-axis denotes the exposure groups, ranging from Group 1 (G1) to Group 4 (G4). The Y-axis conveys the Pearson correlation coefficients determined by Sparse Estimation of Correlations among Microbiomes (SECOM). Precise correlation values are displayed above each data point. The plot exclusively showcases pairwise correlations with at least one non-zero value across the groups.

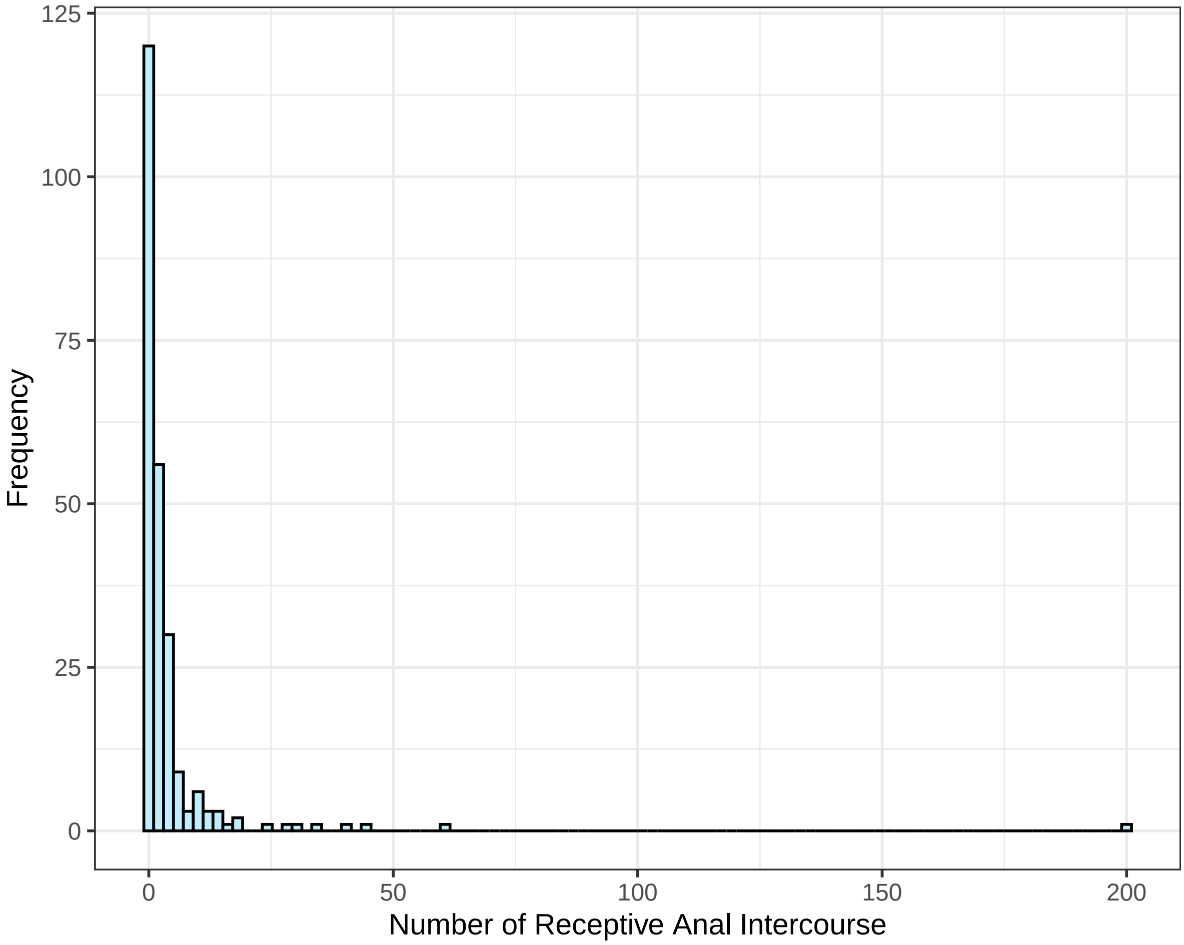

Supplementary Figure 6: **The distribution of the number of receptive anal intercourse among participants.**

3 Supplementary Tables

**Species**	**F statistic**	**Num. d.f.**	**Den. d.f.**	**P-value**
A.muciniphila	0.6	3	212	0.62
A.onderdonkii	0.17	3	212	0.91
Anaerovibrio.spp.	0.0058	3	212	1
B.adolescentis	0.47	3	212	0.71
B.caccae	0.27	3	212	0.85
B.fragilis	1.3	3	212	0.28
B.uniformis	1.4	3	212	0.25
Bacteroides.spp.	0.27	3	212	0.85
Bifidobacterium.spp.	1.4	3	212	0.25
Butyricimonas.spp.	1.2	3	212	0.33
C.celatum	0.86	3	212	0.46
Dehalobacterium.spp.	0.61	3	212	0.61
Lachnobacterium.spp.	0.76	3	212	0.52
Lachnospira.spp.	3.2	3	212	0.02 *
Megasphaera.spp.	0.5	3	212	0.68
Methanobrevibacter.spp.	0.7	3	212	0.55
Odoribacter.spp.	0.4	3	212	0.75
Paraprevotella.spp.	0.85	3	212	0.47
RFN20.spp.	2.8	3	212	0.04 *
Succinivibrio.spp.	1.9	3	212	0.13

Supplementary Table 1: **MANCOVA analysis comparing bias-corrected abundances of DA species with significant plasma cytokine levels.** Abbreviations: Num. d.f. = numerator degrees of freedom; Den. d.f. = denominator degrees of freedom. Significance levels are denoted as follows: * for $p < 0.05$, ** for $p < 0.01$, and *** for $p < 0.001$.

Supplementary Table 2: **Results of the natural effect models [1, 2, 3, 4] for individual mediators.** Sexual exposure groups are the exposure variables, individual biomarkers (cytokines or microbial species) are the mediators, and HIV-1 seroconversion status is the outcome variable. LOR: log odds ratio (natural log base). Trend test p-values were derived using the methodology detailed in Peddada et al. [8]

Mediator	Comparison	LOR	SE	P-Value
	Natural Direct Effect (NDE)			
	G2 – G1	1.99	0.64	0.002
sCD14	G3 – G1	2.59	0.61	0.001
	G4 – G1	3.66	0.69	0.001
	G2 – G1	2.01	0.65	0.002
sCD163	G3 – G1	2.69	0.62	0.001
	G4 – G1	3.76	0.71	0.001
	G2 – G1	-0.62	1.37	0.649
A.muciniphila	G3 – G1	1.57	0.88	0.076
	G4 – G1	3.46	1.37	0.012
	G2 – G1	0.9	1.03	0.382
B.caccae	G3 – G1	2.09	0.95	0.028
	G4 – G1	3.28	1.2	0.006
	G2 – G1	18.57	0.56	0
B.fragilis	G3 – G1	18.54	0.46	0
	G4 – G1	19.92	0.81	0
	G2 – G1	1.88	0.8	0.019
B.uniformis	G3 – G1	2.57	0.77	0.001
	G4 – G1	4.08	0.93	0
	G2 – G1	2.41	0.78	0.002
Bacteroides.spp.	G3 – G1	3.01	0.74	0
	G4 – G1	4.23	0.82	0
	G2 – G1	2.62	1.08	0.015
Butyricimonas.spp.	G3 – G1	3.5	1.05	0.001
	G4 – G1	4.64	1.13	0
	G2 – G1	17.18	0.66	0
Dehalobacterium.spp.	G3 – G1	17.96	0.53	0
	G4 – G1	20.3	1.15	0
	G2 – G1	1.71	1.19	0.152
Methanobrevibacter.spp.	G3 – G1	2.77	1.13	0.014
	G4 – G1	3.94	1.18	0.001
	G2 – G1	2.42	1.09	0.026
Odoribacter.spp.	G3 – G1	3.29	1.06	0.002
	G4 – G1	4.59	1.19	0
	Natural Indirect Effect (NIE)			
	G2 – G1	0.05	0.07	0.431
sCD14	G3 – G1	0.11	0.08	0.175
	G4 – G1	0.22	0.12	0.058
	G2 – G1	0.07	0.08	0.378
sCD163				

Continued on next page

Supplementary Table 2 – continued from previous page

Mediator	Comparison	LOR	SE	P-Value
	G3 – G1	0.04	0.06	0.56
	G4 – G1	0.17	0.12	0.144
	G2 – G1	0.03	0.22	0.887
A.muciniphila	G3 – G1	0.01	0.1	0.894
	G4 – G1	-0.01	0.04	0.896
	G2 – G1	-0.07	0.12	0.597
B.caccae	G3 – G1	0.06	0.12	0.6
	G4 – G1	0.18	0.16	0.256
	G2 – G1	-0.02	0.06	0.77
B.fragilis	G3 – G1	0.09	0.19	0.648
	G4 – G1	0.03	0.08	0.737
	G2 – G1	0.01	0.04	0.737
B.uniformis	G3 – G1	0.07	0.1	0.462
	G4 – G1	0.09	0.12	0.463
	G2 – G1	0.04	0.06	0.433
Bacteroides.spp.	G3 – G1	0.06	0.06	0.345
	G4 – G1	0.18	0.13	0.159
	G2 – G1	-0.03	0.12	0.831
Butyricimonas.spp.	G3 – G1	0.14	0.12	0.224
	G4 – G1	0.36	0.19	0.051
	G2 – G1	0.06	0.12	0.656
Dehalobacterium.spp.	G3 – G1	0.05	0.12	0.69
	G4 – G1	0.06	0.15	0.677
	G2 – G1	0.15	0.16	0.35
Methanobrevibacter.spp.	G3 – G1	0.13	0.14	0.35
	G4 – G1	0.04	0.08	0.589
	G2 – G1	0.02	0.05	0.729
Odoribacter.spp.	G3 – G1	0.05	0.07	0.458
	G4 – G1	0.16	0.16	0.334

References

[1] Theis Lange, Mette Rasmussen, and Lau Caspar Thygesen. Assessing natural direct and indirect effects through multiple pathways. *American journal of epidemiology*, 179(4):513–518, 2014.

[2] Theis Lange, Stijn Vansteelandt, and Maarten Bekaert. A simple unified approach for estimating natural direct and indirect effects. *American journal of epidemiology*, 176(3):190–195, 2012.

[3] Stijn Vansteelandt, Maarten Bekaert, and Theis Lange. Imputation strategies for the estimation of natural direct and indirect effects. *Epidemiologic Methods*, 1(1):131–158, 2012.

[4] Tom Loeys, Beatrijs Moerkerke, Olivia De Smet, Ann Buysse, Johan Steen, and Stijn Vansteelandt. Flexible mediation analysis in the presence of nonlinear relations: beyond the mediation formula. *Multivariate Behavioral Research*, 48(6):871–894, 2013.

[5] Tyler J VanderWeele. Mediation analysis: a practitioner's guide. *Annual review of public health*, 37(1):17–32, 2016.

[6] Johan Steen, Tom Loeys, Beatrijs Moerkerke, and Stijn Vansteelandt. medflex: An r package for flexible mediation analysis using natural effect models. *Journal of Statistical Software*, 76(11):1–46, 2017.

[7] Reuben M Baron and David A Kenny. The moderator–mediator variable distinction in social psychological research: Conceptual, strategic, and statistical considerations. *Journal of personality and social psychology*, 51(6):1173, 1986.

[8] Shyamal D Peddada, Edward K Lobenhofer, Leping Li, Cynthia A Afshari, Clarice R Weinberg, and David M Umbach. Gene selection and clustering for time-course and dose–response microarray experiments using order-restricted inference. *Bioinformatics*, 19(7):834–841, 2003.
